# Relationship between corneal biomechanical properties and structural biomarkers in patients with normal-tension glaucoma: a retrospective study

**DOI:** 10.1186/s12886-018-0673-x

**Published:** 2018-01-15

**Authors:** Keunheung Park, Jonghoon Shin, Jiwoong Lee

**Affiliations:** 1 Department of Ophthalmology, Pusan National University School of Medicine, #179 Gudeok-ro, Seo-gu, Busan, 602-739 South Korea; 2 Biomedical Research Institute, Pusan National University Hospital, Busan, South Korea

**Keywords:** Corneal hysteresis, Ocular response analyser, Normal tension glaucoma

## Abstract

**Background:**

We evaluated the relationships between corneal biomechanical properties and structural parameters in patients with newly diagnosed, untreated normal-tension glaucoma (NTG).

**Methods:**

All subjects were evaluated using an Ocular Response Analyzer (ORA) measuring corneal hysteresis (CH) and the corneal resistance factor (CRF). Central corneal thickness (CCT), Goldmann applanation tonometric (GAT) data, axial length, and the spherical equivalent (SE), were also measured. Confocal scanning laser ophthalmoscopy was performed with the aid of a Heidelberg retina tomograph (HRT III). We sought correlations between HRT parameters and different variables including CCT, CH, and the CRF. Multiple linear regression analysis was performed to identify significant associations between corneal biomechanical properties and optic nerve head parameters.

**Results:**

We enrolled 95 eyes of 95 NTG patients and 93 eyes of 93 normal subjects. CH and the CRF were significantly lower in more advanced glaucomatous eyes (*P* = 0.001, *P* = 0.008, respectively). The rim area, rim volume, linear cup-to-disc ratio (LCDR), and mean retinal nerve fiber layer (RNFL) thickness were significantly worse in more advanced glaucomatous eyes (*P* < 0.001, P < 0.001, P < 0.001, and *P* = 0.001). CH was directly associated with rim area, rim volume, and mean RNFL thickness (*P* = 0.012, *P* = 0.028, and *P* = 0.043) and inversely associated with LCDR (*P* = 0.015), after adjusting for age, axial length, CCT, disc area, GAT data, and SE. However, in normal subjects, there were no significant associations between corneal biomechanical properties and HRT parameters.

**Conclusions:**

A lower CH is significantly associated with a smaller rim area and volume, a thinner RNFL, and a larger LCDR, independent of disc size, corneal thickness, intraocular pressure, and age.

## Background

Glaucoma is characterised by a loss of retinal ganglion cells (RGCs), optic disc cupping, thinning of the retinal nerve fibre layer (RNFL), and associated visual-field defects [1]. Burgoyne suggested that the central pathophysiology of glaucoma is damage to RGC axons within the lamina cribrosa of the optic nerve head [2]. Corneal biomechanical properties, such as corneal hysteresis (CH) and central corneal thickness (CCT) have been found to be significantly associated with individual optic nerve head behaviour and susceptibility to a given level of intraocular pressure (IOP) [3–11]. Indeed, a thinner CCT has been suggested as a risk factor in the development and progression of primary open-angle glaucoma (POAG) [4, 12, 13]. Recent studies have demonstrated that CH, determined with an ocular response analyser (ORA; Reichert Ophthalmic Instruments, Buffalo, New York, USA), offers better corneal biomechanical properties than does CCT [3, 5, 6, 8–10, 14, 15]. Anand et al. reported that worse eye in asymmetric POAG was associated with lower CH, but not with CCT [10]. Moreover, previous longitudinal studies reported that CH may be a more important risk factor related to visual field progression than CCT [3, 9, 11]. Recently, Zhang et al. reported that faster rates of RNFL thickness deterioration was associated with lower CH, but not with CCT [8].

CH is a measure of the viscoelastic damping properties of the corneal tissue [16]. It has been suggested that CH may be related to the biomechanical characteristics of the lamina cribrosa and peripapillary sclera that could affect the susceptibility of the optic nerve head to glaucomatous damage [5, 8, 17]. It also has been suggested that CH could be associated with the biomechanical properties of the optic nerve head [15, 18]. Prata et al. found that eyes with lower CH had a larger cup-to-disc ratio and deeper cup [18]. Khawaja et al. reported that a larger cup-to-disc ratio was associated with lower CH [15]; however, only those with high-tension POAG were included in their study, and the association between structural biomarkers and corneal biomechanical properties in normal-tension glaucoma (NTG) eyes were not assessed [15, 18].

Although POAG with untreated IOP ≤ 21 mmHg is defined as NTG [19], IOP is a major predictor of progression in NTG as well as in high-tension POAG [4, 20–25]. The results of previous studies suggest that CH may be related to the susceptibility of the optic disc to glaucomatous damage induced by IOP [3, 5, 6]. Additionally, CH is significantly lower in NTG patients than in normal subjects [14, 26–28]. Thus, the relationships between structural biomarkers such as optic nerve head parameters and corneal biomechanical properties may differ between the patients with NTG and normal subjects, but no study on the relationship between corneal biomechanical properties and optic nerve head parameters in NTG eyes has yet been reported. Thus, the purpose of this study was to evaluate the relationship between corneal biomechanical properties and optic nerve head parameters in patients with NTG.

## Methods

This was a cross-sectional, comparative study. We retrospectively reviewed the records of glaucoma patients and normal controls who visited the glaucoma clinic of the ophthalmology department of Pusan National University Hospital. We included the normal controls from those who visited our hospital for regular health checkups or for the management of mild ocular diseases such as dry eye syndrome. The study adhered to the tenets of the Declaration of Helsinki and ethics approval was obtained from the Institutional Review Board (IRB) of Pusan National University Hospital (IRB #E-2014121).

The inclusion criteria included age > 18 years, a clear cornea, and no opacity of the ocular media. All eyes had a best-corrected visual acuity (BCVA) ≥ 20/40 and a spherical equivalent refractive error ≤ 5.0 dioptres (D) with astigmatism ≤3.0 D. Exclusion criteria included diabetic retinopathy, corneal abnormalities, uveitis, secondary glaucoma, non-glaucomatous optic neuropathies, or any eye disorder except glaucoma. Patients with histories of previous trauma, ocular surgery, or laser treatment were also excluded. If subjects had already been diagnosed with glaucoma and were using IOP-lowering medication, they were excluded. When both eyes were eligible, one eye was randomly chosen for analysis.

Normal subjects were defined as those with no history of ocular disease, IOP ≤ 21 mmHg, a non-glaucomatous optic nerve head, and a normal visual field. The diagnosis of NTG was based on the following criteria: (1) untreated baseline IOP ≤ 21 mmHg measured from 9 am to 5 pm at intervals of 2 h; (2) an open angle on gonioscopy; (3) characteristic glaucomatous optic nerve head damage; and (4) corresponding visual field loss. Glaucomatous optic neuropathy was defined when one or more of the following criteria was/were met: a cup-to-disc ratio asymmetry ≥0.2; focal or diffuse neuroretinal rim thinning; localised notching; or an RNFL defect congruent with the visual field abnormality [29, 30]. Glaucomatous visual field loss was defined by two or more of the following three criteria: (1) a group of three points on a pattern deviation probability plot with *P* < 0.05, one of which was associated with a *P* value <0.01; (2) a pattern standard deviation with P < 0.05; (3) a Glaucoma Hemifield Test result outside normal limits. All visual field tests had to satisfy the following three criteria in terms of reliability: fixation loss <20%, and false-positive and false-negative rates <15%.

All subjects received a complete ophthalmological examination at the first visit as a routine test of glaucoma clinic, including measurement of IOP performed via Goldmann applanation tonometry (GAT); measurement of axial length and BCVA; slit lamp examination; fundoscopy; and gonioscopy. An Auto Kerato-Refractor (ARK-510A; NIDEK, Hiroishi, Japan) was used to measure the spherical equivalent and for keratometry. CCT was measured with the aid of ultrasonic pachymetry (Micropach; Sonomed, New Hyde Park, NY, USA). Visual field examinations were performed with the 30–2 SITA standard program on a Humphrey 740 automated perimeter (Carl Zeiss Meditec, Dublin, CA, USA). Glaucoma patients were further divided into two subgroups, thus those with early (MD ≥ −6 dB) or moderate-to-advanced (MD < −6 dB) glaucoma.

A trained examiner performed ORA examinations to measure CH, the corneal resistance factor (CRF), corneal-compensated IOP (IOPcc), and Goldmann-correlated IOP (IOPg). The ORA evaluated two applanation pressure points during every test. The first point (P1) was that when the air puff pushed the cornea until it was applanated and the second point (P2) was that when the applanated cornea returned to its normal shape. The difference between these two pressure points (P1 − P2) was defined as the CH. The CRF was derived from a parameter that reflected the general resistance of the cornea to deformation [16]. The IOPcc was the recalculated IOP value using corneal biomechanical information provided by the CH measurement [31]. A good-quality ORA measurement was defined as a measurement evidencing symmetric peak heights, similar widths, and a waveform score > 5.0. At least four good-quality ORA readings were required for inclusion in the study. An experienced investigator judged the quality of all response profiles. To exclude selection bias, we used the best signal value as selected by dedicated software (ORA ver. 3.01).

Confocal scanning laser ophthalmoscopy (CSLO) images were obtained using a Heidelberg Retina Tomograph III (HRT; Heidelberg Engineering, Heidelberg, Germany). Fifteen-degree field-of view scans centred on the optic nerve head were captured and automatically repeated three times at each acquisition. To construct a single composite image, the stack of individual scans was aligned by the software. Any scans that did not meet the quality indices were discarded. An experienced observer reviewed stereoscopic images of the optic nerve head and drew the contour of the optic nerve head on the mean topographic image. The global stereometric parameters calculated by HRT software were exported for further analysis. Of these parameters, we used the rim area, rim volume, linear cup-to-disc ratio, and mean RNFL thickness as the principal HRT outcomes in terms of statistical analyses, because previous studies found significant differences in these parameters between glaucomatous and normal eyes [32, 33]. Also, rim area and rim volume are known to correlate significantly with the development of POAG [34]. To ensure quality control, scans with a global pixel standard deviation ≤40 μm were included in analysis.

All statistical analyses were performed with the SPSS software (ver. 21.0 for Windows; SPSS Inc., Chicago, IL, USA). The normality of the data was checked with the Kolmogorov-Smirnov test. Student’s t-test or the Mann–Whitney U-test was used to compare variables such as age, axial length, GAT, CCT, spherical equivalent, ORA, and HRT parameters between the glaucoma and normal group. Depending on data normality, Pearson’s correlation coefficient or Spearman’s rank correlation coefficient was used to investigate correlations between HRT parameters and multiple variables, including CH, CRF, CCT, GAT, axial length, age, and spherical equivalent. Multiple linear regression analyses with the ENTER method were used to identify significant associations of corneal biomechanical properties and optic nerve head parameters, with adjustment for potential confounding factors. CH was found to be dependent on age, axial length, CCT, IOP, and spherical equivalent [14, 15, 35–37]. Optic nerve head parameters were related to disc size [38, 39]. Thus, age, axial length, CCT, GAT, spherical equivalent, and disc size were also entered into the multiple linear regression model as explanatory variables, together with corneal parameters. *P*-values <0.05 were considered to reflect statistical significance.

## Results

95 patients (95 eyes) with NTG and 93 subjects (93 eyes) in the normal healthy control group were included in this study. Ophthalmic and demographic characteristics are summarised in Table 1. There were no significant differences in axial length, GAT, CCT, spherical equivalent, IOPg, or IOPcc between patients with NTG and normal controls, while CH and the CRF were significantly lower in eyes with more advanced glaucoma (*P* = 0.001 and *P* = 0.008, respectively). Visual field parameters including mean deviation (MD), pattern standard deviation (PSD), and visual field index (VFI) were significantly worse in eyes with more advanced glaucoma (all *P* < 0.001). HRT parameters, including linear cup-to-disc ratio, rim area, rim volume, and mean RNFL thickness, were also significantly worse in the more advanced glaucomatous eyes (*P* < 0.001, *P* < 0.001, *P* < 0.001, and *P* = 0.001, respectively).

**Table 1:** Demographic and clinical characteristics, including corneal biomechanical properties and optic nerve head parameters

	Normal controls	NTG patients		*P* value
		Early	Advanced	
Subjects (n)	93	48	47	
Age (years)	56.35 ± 10.46	53.92 ± 12.03	62.38 ± 11.96	<0.001^a^
Gender (male/female)	52 / 41	22 / 26	32 / 15	0.009^b^
Axial length (mm)	23.79 ± 0.88	23.78 ± 0.91	23.96 ± 1.24	0.590^c^
GAT (mmHg)	14.97 ± 2.53	15.10 ± 3.18	15.17 ± 2.99	0.874^a^
CCT (μm)	550.84 ± 26.84	551.29 ± 33.45	544.66 ± 33.62	0.465^c^
Spherical equivalent (diopter)	−0.50 ± 1.68	−0.43 ± 1.61	−0.59 ± 1.95	0.891^a^
Visual field				
MD (dB)	−1.57 ± 1.57	−3.10 ± 1.59	−12.63 ± 5.63	<0.001^a^
VFI (%)	98.67 ± 1.74	94.21 ± 4.37	67.17 ± 21.49	<0.001^a^
PSD (dB)	1.93 ± 0.80	3.84 ± 2.36	9.72 ± 3.97	<0.001^a^
ORA parameters (mm Hg)				
CH	10.83 ± 1.60	10.56 ± 1.44	9.78 ± 1.52	0.001^c^
CRF	10.67 ± 1.88	10.16 ± 1.84	9.67 ± 1.34	0.008^a^
IOPg	15.15 ± 3.96	14.21 ± 3.60	14.79 ± 3.51	0.373^c^
IOPcc	15.22 ± 3.70	14.69 ± 3.06	16.05 ± 3.93	0.183^c^
HRT parameters				
Linear cup-to-disc ratio	0.62 ± 0.15	0.69 ± 0.12	0.72 ± 0.13	<0.001^a^
Rim area (mm^2^)	1.57 ± 0.47	1.10 ± 0.28	0.98 ± 0.28	<0.001^a^
Rim volume (mm^3^)	0.38 ± 0.19	0.26 ± 0.12	0.20 ± 0.10	<0.001^a^
Mean RNFL thickness (mm)	0.23 ± 0.09	0.20 ± 0.08	0.16 ± 0.11	0.001^a^

^a^Kruskal–Wallis test, ^b^ χ^2^ test, ^c^ one-way ANOVA test

*CCT* Central corneal thickness, *CH* Corneal hysteresis, *CRF* Corneal resistance factor, *GAT* Goldmann applanation tonometry, *HRT* Heidelberg Retina Tomograph, *IOPg* Goldmann-correlated intraocular pressure, *IOPcc* Corneal-compensated intraocular pressure, *MD* Mean deviation, *NTG* Normal tension glaucoma, *0* Ocular Response Analyzer, *PSD* Pattern standard deviation, *RNFL* Retinal nerve fibre layer, *VFI* Visual field index

**Fig. 1:**
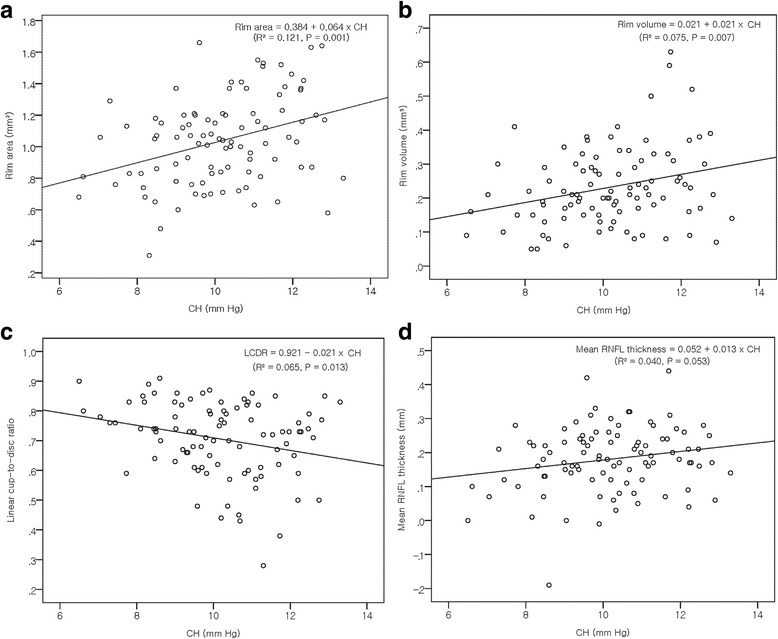
Scatter plot showing the influence of corneal hysteresis (CH) on various ONH parameters in patients with NTG. **a** Corneal hysteresis was positively correlated with rim area (*r* = 0.347, *P* = 0.001), (**b**) rim volume (*r* = 0.274, *P* = 0.007), (**c**) negatively correlated with linear cup-to-disc ratio (*r* = −0.254, *P* = 0.013), and (**d**) positively correlated with mean retinal nerve fibre layer thickness (*r* = 0.199, *P* = 0.053) in NTG patients. (CH= corneal hysteresis, LCDR= linear cup-to-disc ratio, ONH= optic nerve head, NTG= normal tension glaucoma, RNFL= retinal nerve fibre layer thickness)

Pearson’s correlation coefficient or Spearman’s rank correlation coefficient for ophthalmic variables and optic nerve head parameters in patients with NTG and normal subjects are summarized in Tables 2 and 3. CH was positively correlated with rim area and rim volume, and negatively correlated with linear cup-to-disc ratio in NTG patients (*P* = 0.001, *P* = 0.007, and *P* = 0.013, respectively) (Fig. 1). CRF was positively correlated with rim area in NTG patients (*P* = 0.027). CCT was positively correlated with rim area and negatively correlated with linear cup-to-disc ratio in NTG patients (*P* = 0.012 and *P* = 0.019, respectively). Age correlated negatively with rim volume and mean RNFL thickness in both NTG patients (*P* = 0.001 and *P* < 0.001, respectively) and normal controls (*P* = 0.003 and *P* < 0.001, respectively). Disc area was positively correlated with linear cup-to-disc ratio in NTG patients (*P* < 0.001), and linear cup-to-disc ratio, rim area in normal controls (*P* < 0.001). Disc area was negatively correlated with mean RNFL thickness in both NTG patients and normal controls (*P* < 0.001 and *P* = 0.001, respectively). However, there was no significant correlation between corneal biomechanical properties and HRT parameters in the normal controls.

**Table 2:** Pearson correlation coefficients for ophthalmic variables and ONH parameters in NTG patients

	Linear cup-to-disc ratio		Rim area		Rim volume		Mean RNFL thickness	
	r	*P* value	r	*P* value	r	*P* value	r	*P* value
Age	0.187^a^	0.070	−0.061^a^	0.556	−0.324^a^	0.001	−0.476^a^	<0.001
Axial length	−0.105	0.310	0.068	0.512	0.161	0.118	0.240	0.019
Disc area	0.615^a^	<0.001	0.072^a^	0.490	−0.170^a^	0.099	−0.450^a^	<0.001
SE	0.178^a^	0.084	−0.033^a^	0.751	−0.265^a^	0.009	−0.361^a^	<0.001
CCT	−0.239	0.019	0.256	0.012	0.190	0.066	0.159	0.123
CH	−0.254	0.013	0.347	0.001	0.274	0.007	0.199	0.053
CRF	−0.119	0.250	0.227	0.027	0.174	0.092	0.149	0.150
GAT	0.042	0.683	−0.053	0.608	−0.013	0.897	0.067	0.521

^a^Spearman’s rho

*CCT* Central corneal thickness, *CH* Corneal hysteresis, *CRF* Corneal resistance factor, *GAT* Goldmann applanation tonometry, *HRT* Heidelberg retina tomograph, *NTG* Normal tension glaucoma, *ONH* Optic nerve head, *RNFL* Retinal nerve fibre layer, *SE* Spherical equivalent

**Table 3:** Pearson correlation coefficients for ophthalmic variables and ONH parameters in normal subjects

	Linear cup-to-disc ratio		Rim area		Rim volume		Mean RNFL thickness	
	r	*P* value	r	*P* value	r	*P* value	r	*P* value
Age	0.166^a^	0.111	−0.133^a^	0.205	−0.307^a^	0.003	−0.403^a^	<0.001
Axial length	−0.223	0.032	0.080	0.445	0.151	0.150	0.167	0.110
Disc area	0.363^a^	<0.001	0.371^a^	<0.001	−0.015^a^	0.890	−0.348^a^	0.001
SE	0.232^a^	0.025	−0.125^a^	0.234	−0.300^a^	0.003^†^	−0.371^a^	<0.001
CCT	−0.071	0.501	−0.022	0.833	−0.002	0.987	0.100	0.339
CH	0.016	0.878	0.022	0.832	0.034	0.745	0.024	0.819
CRF	0.014	0.895	0.001	0.996	0.018	0.863	0.023	0.824
GAT	−0.068	0.520	−0.090	0.393	−0.022	0.834	0.053	0.617

^a^Spearman’s rho

*CCT* Central corneal thickness, *CH* Corneal hysteresis, *CRF* Corneal resistance factor, *GAT* Goldmann applanation tonometry, *HRT* Heidelberg retina tomograph, *ONH* optic nerve head, *RNFL* Retinal nerve fibre layer, *SE* Spherical equivalent

Multiple linear regression analyses were performed to investigate the parameters that may affect the optic nerve head parameters in both groups (Tables 4 and 5). Regression models were constructed with a HRT parameter as the outcome variable, and CH or the CRF as a covariate. Additionally, each model was adjusted for the following covariates: age, axial length, spherical equivalent, GAT, CCT, and disc area. CH was directly associated with rim area, rim volume, and mean RNFL thickness, and inversely associated with linear cup-to-disc ratio in NTG patients. Neither CCT nor the CRF was associated with the HRT parameter in the adjusted analyses in NTG patients. The corneal biomechanical properties were not associated with any HRT parameter in the adjusted analyses in normal controls. The coefficients for CH, the CRF, and CCT in these models are presented in Tables 4 and 5.

**Table 4:** Multiple linear regression analysis for association between corneal parameters and ONH parameters in NTG patients

	Linear cup to disc ratio			Rim area			Rim volume			Mean RNFL thickness		
	ß	95% CI	*P* value	ß	95% CI	*P* value	ß	95% CI	*P* value	ß	95% CI	*P* value
CH	−0.019	−0.034 ~ −0.004	0.015	0.054	0.012~ 0.096	0.012	0.019	0.002 ~ 0.036	0.028	0.013	0.000 ~ 0.025	0.043
CCT	0.000	−0.001 ~ 0.000	0.259	0.001	−0.001 ~ 0.003	0.207	0.000	−0.001 ~ 0.001	0.782	0.000	−0.001 ~ 0.000	0.693
CRF	−0.011	−0.027 ~ 0.005	0.189	0.044	−0.001 ~ 0.089	0.057	0.016	−0.002 ~ 0.034	0.090	0.011	−0.002 ~ 0.024	0.095
CCT	−0.001	−0.001 ~ 0.000	0.082	0.002	0.000 ~ 0.004	0.096	0.000	−0.001 ~ 0.001	0.455	0.000	−0.001 ~ 0.001	0.892

Table contains the results for eight regression models. The outcome variables are listed in the first row. Each model includes corneal hysteresis or corneal resistance factor as a covariate. Additionally, each model was adjusted for the following covariates: age, axial length, spherical equivalent, Goldmann applanation tonometry, CCT, and disc area

*CCT* Central corneal thickness, *CH* Corneal hysteresis, *CI* Confidence interval, *CRF* Corneal resistance factor, *NTG* Normal tension glaucoma, *ONH* Optic nerve head, *RNFL* Retinal nerve fibre layer

**Table 5:** Multiple linear regression analysis for association between corneal parameters and ONH parameters in normal controls

	Linear cup to disc ratio			Rim area			Rim volume			Mean RNFL thickness		
	ß	95% CI	*P* value	ß	95% CI	*P* value	ß	95% CI	*P* value	ß	95% CI	*P* value
CH	−0.007	−0.029 ~ 0.014	0.503	0.030	−0.035 ~ 0.096	0.358	0.018	−0.010 ~ 0.045	0.205	0.004	−0.007 ~ 0.016	0.461
CCT	0.000	−0.001 ~ 0.001	0.857	−0.001	−0.004 ~ 0.003	0.760	−0.001	−0.002 ~ 0.001	0.348	0.000	−0.001 ~ 0.001	0.907
CRF	−0.001	−0.020 ~ 0.018	0.913	0.010	−0.048 ~ 0.068	0.727	0.012	−0.013 ~ 0.036	0.352	0.003	−0.007 ~ 0.013	0.546
CCT	0.000	−0.001 ~ 0.001	0.942	0.000	−0.004 ~ 0.004	0.962	−0.001	−0.002 ~ 0.001	0.432	0.000	−0.001 ~ 0.001	0.956

Table contains the results for eight regression models. The outcome variables are listed in the first row. Each model includes corneal hysteresis or corneal resistance factor as a covariate. Additionally, each model was adjusted for the following covariates: age, axial length, spherical equivalent, Goldmann applanation tonometry, CCT, and disc area

*CCT* Central corneal thickness, *CH* Corneal hysteresis, *CI* Confidence interval, *CRF* Corneal resistance factor, *NTG* Normal tension glaucoma, *ONH* Optic nerve head, *RNFL* Retinal nerve fibre layer

The results of multiple linear regression analyses of the associations among corneal parameters and visual field parameters in NTG patients are presented in Table 6. The models were constructed using visual field parameters (MD, VFI) as outcome variables, and CH, CRF, age, axial length, spherical equivalent, GAT, CCT, and disc area as covariates. Of the corneal parameters, CH and CRF had significant associations with visual field parameters. CH and CRF were directly associated with MD and VFI. In normal subjects, no independent parameters were associated with any visual field parameter.

**Table 6:** Multiple linear regression analysis for association between corneal parameters and visual field parameters in NTG patients

	Mean deviation (dB)			Visual Field Index (%)		
	ß	95% CI	*P* value	ß	95% CI	*P* value
CH	1.237	0.43478	0.006	3.705	1.42086	0.011
CCT	−0.015	0.02065	0.468	−0.033	0.06748	0.6225
CRF	1.221	0.46078	0.010	4.001	1.49434	0.009
CCT	−0.010	0.02027	0.609	−0.026	0.06573	0.68913

Table contains the results of two regression models. The outcome variables are listed in the first row. Models were constructed with ONH parameters as the dependent variable and corneal hysteresis, corneal resistance factor, CCT, age, axial length, spherical equivalent, Goldmann applanation tonometry, and disc area as explanatory variables

*CCT* Central corneal thickness, *CH* Corneal hysteresis, *CI* Confidence interval, *CRF* Corneal resistance factor, *GAT* Goldmann applanation tonometry, *NTG* Normal tension glaucoma, *ONH* Optic nerve head, *RNFL* Retinal nerve fibre layer

## Discussion

We evaluated the relationships between corneal biomechanical properties and optic nerve head parameters in patients with untreated and newly diagnosed NTG. CH was positively correlated with rim area and rim volume, and negatively with the linear cup-to-disc ratio in NTG patients. CRF was positively correlated with rim area in NTG patients. CCT was positively correlated with rim area and negatively correlated with linear cup-to-disc ratio in NTG patients. However, when age, axial length, CCT, disc area, GAT, spherical equivalent, and CRF or CH were considered as independent variables in multiple regression analysis, lower CH alone was significantly associated with smaller rim area and volume, thinner RNFL thickness, and larger linear cup-to-disc ratio in NTG patients. The CH, CRF, and CCT were not associated with any HRT parameter in univariate and multivariate analyses in normal controls.

Thinner CCT was a significant progression factor in the Early Manifest Glaucoma Trial (EMGT) [4]. The CCT may be indicative of anatomical structure, and may reflect the elasticity and distensibility of ocular tissues [4, 11]. Jonas et al. found that CCT was significantly correlated with rim area [40]. Congdon et al. reported that a thinner CCT was correlated with a greater cup-to-disc ratio in 230 subjects [11]. In accordance with previous studies, we also found that CCT was significantly correlated with rim area and linear cup-to-disc ratio. However, CCT was not associated with optic nerve head parameter when CH or CRF was entered in multiple regression analysis at the same time. The CH alone remained significantly associated with optic nerve head parameters in NTG patients after adjusting for age, axial length, CCT, disc size, GAT, and spherical equivalent.

Congdon et al. found that lower CH, but not CCT, was associated with visual field progression in 194 subjects with POAG or suspected glaucoma using multivariate generalised estimation equation models [11]. Anand et al. found that worse eyes were associated with lower CH in 117 POAG patients using the Advanced Glaucoma Intervention Study (AGIS) score, while neither the mean CCT nor GAT was asymmetric between the worse and better eyes [10]. De Moraes et al. found that lower CH was associated with progression of visual field damage in a multivariable model, whereas CCT was not [9]. In a prospective observational cohort study, Medeiros et al. reported that lower CH was associated with a faster decline in VFI and CH explained a larger proportion of the variation in slope of VFI change than CCT [3]. Zhang et al. found that lower CH was associated with faster thinning of the RNFL whereas CCT was not [8].

We found that NTG patients had lower CH than the normal group. These results are consistent with the earlier studies which found that CH is significantly lower in NTG patients than in normal subjects [14, 26–28]. Morita et al. found that the IOPcc of NTG eyes is significantly higher than that of normal eyes [26]. Ehrlich et al. reported that the IOPcc in patients with NTG eyes was greater than the GAT and that the difference between IOPcc and GAT was larger in patients with NTG than in patients with high-tension POAG or normal subjects [41].

The findings in this study indicate that CH has a greater influence on structural biomarkers than does CCT in NTG patients. Our findings are generally consistent with the earlier studies that examined the relationship between corneal biomechanical properties and optic nerve head topographic parameters [15, 17, 18]. Prata et al. found that CH was the only corneal parameter significantly associated with both linear cup-to-disc ratio and mean cup depth after controlling for age, race, IOP, disc area, and CCT in 42 patients with high-tension POAG [18]. However, they did not report the relationship between corneal biomechanical properties and HRT parameters in NTG patients [18]. Recently, a population-based study reported that CH was significantly associated with anatomical quantitative features of the optic nerve head in the same direction as that seen in glaucoma after adjusting for GAT, CCT, and possible confounders [15]. However, the study lacks specificity of the association with a particular form of glaucoma and a definite relationship of CH with optic nerve head parameters in NTG patients was not established. Bochmann et al. demonstrated that CH in POAG patients with acquired pit was significantly lower than in those patients without structural changes in the optic disc, whereas CCT did not differ between the groups [17]. Two studies have reported that lower CH was associated with thinner RNFL thickness in patients with established or suspected glaucoma [42, 43]; however, the relationship was not statistically significant in a multivariable model after adjusting for possible confounders. These two studies differed from our study in that patients in the previous studies were treated with IOP-lowering therapy, which may affect the relationship between CH and structural markers of glaucoma [43]. The authors of the previous study did not report a particular type of glaucoma; thus, an association with NTG may not have been detected.

There was no statistically significant relationship between corneal factors (CH, the CRF, and CCT) and HRT parameters in the normal group. The relationship between CH and optic nerve head parameters observed in the present study was found only in NTG patients. This suggests that biomechanical changes in ocular tissue occurred in patients with NTG. Our findings are consistent with those of previous studies that investigated the relationship between CCT and the optic nerve head in an elderly normal population [44]. Hawker et al. found no significant correlation between CCT and any global optic nerve head parameters in 690 eyes with normal visual fields [44]. Similarly, a prospective experimental study reported that a normal control group did not show any association between CH and mean cup depth during IOP elevation, whereas a significant association was evident in the glaucoma group [5]. It is possible that the range of HRT parameters in the normal group may be narrow, which may explain the lack of association between corneal biomechanical properties and optic nerve head parameters in this study.

Many researchers have suggested that CH may be related to the biomechanical characteristics of the sclera and lamina cribrosa [5, 8, 17, 25]. Corneal stroma and sclera develop from mesoderm [36]. Furthermore, the collagen of the corneal stroma is continuous with the sclera and lamina cribrosa, despite the differences in embryonic development between the cornea and lamina cribrosa [45, 46]. Thus, it seems possible that the cornea, sclera, and lamina cribrosa share similar biomechanical characteristics and CH represents the response of the entire eye wall, rather than of the cornea alone [5, 36, 47]. This is consistent with studies that reported that eyes with lower CH had a significantly greater decrease in axial length after trabeculectomy and high myopes had lower CH than emmetropes [36, 48]. Johnson et al. demonstrated that the pressure–volume curves of a corneal-scleral shell had the same shape as the pressure–volume curve of a whole globe [7].

Based on previous studies that reported an association between CH and optic nerve head surface compliance, it has been suggested that CH may represent an indirect measure of lamina cribrosa compliance [5, 6, 17, 47]. Wells et al. found that CH was significantly correlated with mean cup depth increase during transient elevation of IOP in glaucoma patients [5]. Prata et al. reported that a lower CH was correlated with a greater change in optic nerve head parameters after IOP reduction in 42 patients with POAG [6]. Lesk et al. demonstrated that a thinner CCT was associated with greater shallowing of the optic cup following IOP reduction in patients with POAG and ocular hypertension [47].

Burgoyne et al. suggested that IOP-related stress and strain on the optic nerve head depended on the biomechanical properties of the optic nerve head, which are associated primarily with the biomechanical properties of the lamina cribrosa, scleral canal, and peripapillary sclera [49]. Changes in the extracellular matix (ECM) of the lamina cribrosa in human and monkey eyes with glaucoma have been reported [50, 51]. Downs et al. found alterations in the viscoelastic properties of the peripapillary sclera of monkey eyes exposed to moderate, short-term, chronic IOP elevation [52]. Girard et al. reported scleral stiffening of monkey eyes in response to moderate IOP elevation [53]. They suggested that these biomechanical changes may be the result of scleral ECM remodelling [52, 53]. Thus, lower CH may be related to increased susceptibility of the optic disc to glaucomatous damage, induced by IOP elevation [3, 5, 6]. IOP increments of the order of 90 mmHg for squeezing of lids, 10 mmHg for eye turned to the side, and 10 mmHg for blinking were recorded in a study by Coleman and Trokel [54]. Patients with lower CH may be less able to damp these brief IOP fluctuations sufficiently when they squeeze, blink, or rub their eyes [7]. EMGT and the Collaborative Normal-Tension Glaucoma Study reported that progression occurred in some eyes with NTG, despite lowering of IOP [20, 23]. The optic nerve head in NTG eyes with lower CH may be more vulnerable to glaucomatous damage even when the GAT value is in the normal range [18]. This may explain partially the association of CH and structural biomarkers in NTG eyes. Given that the multiple regression models in the current investigation were adjusted for GAT, CH may be an IOP-independent risk factor for glaucoma related to the constitution of whole globe wall [9, 11].

Tsikripis et al. found prostaglandin analogue instillation was associated with an increase in CH [55]. An experimental study reported that latanoprost may upregulate matrix metalloproteinases, which can originate degeneration of ECM components [56]. However, we evaluated corneal biomechanical properties and optic nerve head parameter in newly diagnosed untreated cases of NTG to minimise the influence of long term use of prostaglandins on biomechanical properties of cornea.

Our study had certain limitations. First, the sample size of the study population was relatively modest. Second, all subjects included in this study were Asian; the relationship between biomechanical properties of cornea and structural measures may differ in other populations because CH differs by ethnicity [57]. Third, glaucoma severity was not assessed as an independent variable which may influence the impact of corneal biomechanical properties on HRT parameters. Fourth, the study was performed cross-sectionally. Thus, we were unable to determine whether the relationship between CH and HRT parameter in patients with NTG reflects a cause or an effect.

## Conclusions

We found that lower corneal hysteresis was associated with smaller rim area and volume, larger linear cup-to-disc ratio, and thinner mean RNFL thickness in newly diagnosed untreated NTG patients after adjustment for age, axial length, corneal thickness, disc size, IOP, and spherical equivalent. The results of this study highlight the importance of corneal biomechanical properties on changes in the optic nerve head in NTG patients and may improve our understandings of the pathophysiological mechanism(s) involved in the development of glaucomatous optic neuropathy.

## Abbreviations

BCVA: Best-corrected visual acuity
CCT: Central corneal thickness
CH: Corneal hysteresis
CI: Confidence interval
CRF: Corneal resistant factor
D: Diopter
ECM: Extra cellular matix
EMGT: Early manifest glaucoma trial
GAT: GOLDMANN applanation tonometry
HRT: Heidelberg retina tomograph
IOP: Intraocular pressure
IOPcc: Corneal-compensated intraocular pressure
IOPg: Goldmann-correlated intraocular pressure
MD: Mean deviation
NTG: Normal-tension glaucoma
ORA: Ocular response analyser
POAG: Primary open-angle glaucoma
PSD: Pattern standard deviation
RGC: Retinal ganglion cell
RNFL: Retinal nerve fibre layer
SE: Spherical equivalent
VFI: Visual field index

## References

[1] Weinreb RN, Khaw PT (2004). Primary open-angle glaucoma. Lancet.

[2] Burgoyne CFA (2011). Biomechanical paradigm for axonal insult within the optic nerve head in aging and glaucoma. Exp Eye Res.

[3] Medeiros FA, Meira-Freitas D, Lisboa R, Kuang T-M, Zangwill LM, Weinreb RN (2013). Corneal hysteresis as a risk factor for glaucoma progression: a prospective longitudinal study. Ophthalmology.

[4] Leske MC, Heijl A, Hyman L, Bengtsson B, Dong L, Yang Z (2007). Predictors of long-term progression in the early manifest glaucoma trial. Ophthalmology.

[5] Wells AP, Garway-Heath DF, Poostchi A, Wong T, Chan KCY, Sachdev N (2008). Corneal hysteresis but not corneal thickness correlates with optic nerve surface compliance in glaucoma patients. Invest Ophthalmol Vis Sci.

[6] Prata TS, Lima VC, de Moraes CGV, Guedes LM, Magalhães FP, Teixeira SH (2011). Factors associated with topographic changes of the optic nerve head induced by acute intraocular pressure reduction in glaucoma patients. Eye Lond Engl..

[7] Johnson CS, Mian SI, Moroi S, Epstein D, Izatt J, Afshari NA (2007). Role of corneal elasticity in damping of intraocular pressure. Invest Ophthalmol Vis Sci.

[8] Zhang C, Tatham AJ, Abe RY, Diniz-Filho A, Zangwill LM, Weinreb RN, et al. Corneal hysteresis and progressive retinal nerve fiber layer loss in glaucoma. Am J Ophthalmol. 2016; 10.1016/j.ajo.2016.02.034 PMC5758050 26949135

[9] De Moraes CVG, Hill V, Tello C, Liebmann JM, Ritch R (2012). Lower corneal hysteresis is associated with more rapid glaucomatous visual field progression. J Glaucoma.

[10] Anand A, De Moraes CGV, Teng CC, Tello C, Liebmann JM, Ritch R (2010). Corneal hysteresis and visual field asymmetry in open angle glaucoma. Invest Ophthalmol Vis Sci.

[11] Congdon NG, Broman AT, Bandeen-Roche K, Grover D, Quigley HA (2006). Central corneal thickness and corneal hysteresis associated with glaucoma damage. Am J Ophthalmol.

[12] Medeiros FA, Sample PA, Zangwill LM, Bowd C, Aihara M, Weinreb RN (2003). Corneal thickness as a risk factor for visual field loss in patients with preperimetric glaucomatous optic neuropathy. Am J Ophthalmol.

[13] Gordon MO, Beiser JA, Brandt JD, Heuer DK, Higginbotham EJ, Johnson CA (2002). The ocular hypertension treatment study: baseline factors that predict the onset of primary open-angle glaucoma. Arch Ophthalmol.

[14] Shin J, Lee J-W, Kim E-A, Caprioli J (2015). The effect of corneal biomechanical properties on rebound tonometer in patients with normal-tension glaucoma. Am J Ophthalmol.

[15] Khawaja AP, Chan MPY, Broadway DC, Garway-Heath DF, Luben R, Yip JLY (2014). Corneal biomechanical properties and glaucoma-related quantitative traits in the EPIC-Norfolk eye study. Invest Ophthalmol Vis Sci.

[16] Luce DA (2005). Determining in vivo biomechanical properties of the cornea with an ocular response analyzer. J Cataract Refract Surg.

[17] Bochmann F, Ang GS, Azuara-Blanco A (2008). Lower corneal hysteresis in glaucoma patients with acquired pit of the optic nerve (APON). Graefes Arch Clin Exp Ophthalmol.

[18] Prata TS, Lima VC, Guedes LM, Biteli LG, Teixeira SH, de Moraes CG (2012). Association between corneal biomechanical properties and optic nerve head morphology in newly diagnosed glaucoma patients. Clin Exp Ophthalmol.

[19] Kim C, Seong GJ, Lee N, Song K, Society KG, Group NS (2011). Prevalence of primary open-angle glaucoma in central South Korea: the Namil study. Ophthalmology.

[20] Heijl A, Leske MC, Bengtsson B, Hyman L, Bengtsson B, Hussein M (2002). Reduction of intraocular pressure and glaucoma progression: results from the early manifest glaucoma trial. Arch Ophthalmol.

[21] Jeong JH, Park KH, Jeoung JW, Kim DM (2014). Preperimetric normal tension glaucoma study: long-term clinical course and effect of therapeutic lowering of intraocular pressure. Acta Ophthalmol.

[22] Kim KE, Jeoung JW, Kim DM, Ahn SJ, Park KH, Kim SH (2015). Long-term follow-up in preperimetric open-angle glaucoma: progression rates and associated factors. Am J Ophthalmol.

[23] Anderson D, Drance SM, Schulzer M (1998). The effectiveness of intraocular pressure reduction in the treatment of normal-tension glaucoma. Am J Ophthalmol.

[24] Kim M, Kim DM, Park KH, Kim T-W, Jeoung JW, Kim SH (2013). Intraocular pressure reduction with topical medications and progression of normal-tension glaucoma: a 12-year mean follow-up study. Acta Ophthalmol.

[25] Shigeeda T, Tomidokoro A, Araie M, Koseki N, Yamamoto S (2002). Long-term follow-up of visual field progression after trabeculectomy in progressive normal-tension glaucoma. Ophthalmology.

[26] Morita T, Shoji N, Kamiya K, Fujimura F, Shimizu K (2012). Corneal biomechanical properties in normal-tension glaucoma. Acta Ophthalmol.

[27] Grise-Dulac A, Saad A, Abitbol O, Febbraro J-L, Azan E, Moulin-Tyrode C (2012). Assessment of corneal biomechanical properties in normal tension glaucoma and comparison with open-angle glaucoma, ocular hypertension, and normal eyes. J Glaucoma.

[28] Kaushik S, Pandav SS, Banger A, Aggarwal K, Gupta A (2012). Relationship between corneal biomechanical properties, central corneal thickness, and intraocular pressure across the spectrum of glaucoma. Am J Ophthalmol.

[29] Jonas JB, Budde WM, Panda-Jonas S (1999). Ophthalmoscopic evaluation of the optic nerve head. Surv Ophthalmol.

[30] Foster PJ, Buhrmann R, Quigley HA, Johnson GJ (2002). The definition and classification of glaucoma in prevalence surveys. Br J Ophthalmol.

[31] Martinez-de-la-Casa JM, Garcia-Feijoo J, Fernandez-Vidal A, Mendez-Hernandez C, Garcia-Sanchez J (2006). Ocular response analyzer versus Goldmann applanation tonometry for intraocular pressure measurements. Invest Ophthalmol Vis Sci.

[32] Medeiros FA, Vizzeri G, Zangwill LM, Alencar LM, Sample PA, Weinreb RN (2008). Comparison of retinal nerve fiber layer and optic disc imaging for diagnosing glaucoma in patients suspected of having the disease. Ophthalmology.

[33] Okimoto S, Yamashita K, Shibata T, Kiuchi Y (2015). Morphological features and important parameters of large optic discs for diagnosing glaucoma. PLoS One.

[34] Zangwill LM, Weinreb RN, Beiser JA, Berry CC, Cioffi GA, Coleman AL (2005). Baseline topographic optic disc measurements are associated with the development of primary open-angle glaucoma: the confocal scanning laser ophthalmoscopy ancillary study to the ocular hypertension treatment study. Arch Ophthalmol.

[35] Kotecha A, Elsheikh A, Roberts CR, Zhu H, Garway-Heath DF (2006). Corneal thickness-and age-related biomechanical properties of the cornea measured with the ocular response analyzer. Invest Ophthalmol Vis Sci.

[36] Wong Y-Z, Lam AKC (2015). The roles of cornea and axial length in corneal hysteresis among emmetropes and high myopes: a pilot study. Curr Eye Res.

[37] Bueno-Gimeno I, España-Gregori E, Gene-Sampedro A, Lanzagorta-Aresti A, Piñero-Llorens DP (2014). Relationship among corneal biomechanics, refractive error, and axial length. Optom Vis Sci Off Publ Am Acad Optom.

[38] Garway-Heath DF, Hitchings RA (1998). Quantitative evaluation of the optic nerve head in early glaucoma. Br J Ophthalmol.

[39] Garway-Heath DF, Ruben ST, Viswanathan A, Hitchings RA (1998). Vertical cup/disc ratio in relation to optic disc size: its value in the assessment of the glaucoma suspect. Br J Ophthalmol.

[40] Jonas JB, Stroux A, Velten I, Juenemann A, Martus P, Budde WM (2005). Central corneal thickness correlated with glaucoma damage and rate of progression. Invest Ophthalmol Vis Sci.

[41] Ehrlich JR, Radcliffe NM, Shimmyo M (2012). Goldmann applanation tonometry compared with corneal-compensated intraocular pressure in the evaluation of primary open-angle glaucoma. BMC Ophthalmol.

[42] Mansouri K, Leite MT, Weinreb RN, Tafreshi A, Zangwill LM, Medeiros FA (2012). Association between corneal biomechanical properties and glaucoma severity. Am J Ophthalmol.

[43] Vu DM, Silva FQ, Haseltine SJ, Ehrlich JR, Radcliffe NM (2013). Relationship between corneal hysteresis and optic nerve parameters measured with spectral domain optical coherence tomography. Graefes Arch Clin Exp Ophthalmol Albrecht Von Graefes Arch Für Klin Exp Ophthalmol.

[44] Hawker MJ, Edmunds MR, Vernon SA, Hillman JG, MacNab HK (2009). The relationship between central corneal thickness and the optic disc in an elderly population: the Bridlington eye assessment project. Eye Lond Engl.

[45] McBrien NA, Gentle A (2003). Role of the sclera in the development and pathological complications of myopia. Prog Retin Eye Res.

[46] Jonas JB, Holbach L (2005). Central corneal thickness and thickness of the lamina cribrosa in human eyes. Invest Ophthalmol Vis Sci.

[47] Lesk MR, Hafez AS, Descovich D (2006). Relationship between central corneal thickness and changes of optic nerve head topography and blood flow after intraocular pressure reduction in open-angle glaucoma and ocular hypertension. Arch Ophthalmol Chic Ill 1960.

[48] Huang C, Zhang M, Huang Y, Chen B, Lam DSC, Congdon N (2012). Corneal hysteresis is correlated with reduction in axial length after trabeculectomy. Curr Eye Res.

[49] Burgoyne CF, Downs JC, Bellezza AJ, Suh J-KF, Hart RT. The optic nerve head as a biomechanical structure: a new paradigm for understanding the role of IOP-related stress and strain in the pathophysiology of glaucomatous optic nerve head damage. Prog Retin Eye Res 2005;24:39–73. 10.1016/j.preteyeres.2004.06.001 15555526

[50] Quigley HA, Brown A, Dorman-Pease ME (1991). Alterations in elastin of the optic nerve head in human and experimental glaucoma. Br J Ophthalmol.

[51] Pena JD, Agapova O, Gabelt BT, Levin LA, Lucarelli MJ, Kaufman PL (2001). Increased elastin expression in astrocytes of the lamina cribrosa in response to elevated intraocular pressure. Invest Ophthalmol Vis Sci.

[52] Downs JC, Suh JF, Thomas KA, Bellezza AJ, Hart RT, Burgoyne CF (2005). Viscoelastic material properties of the peripapillary sclera in normal and early-glaucoma monkey eyes. Invest Ophthalmol Vis Sci.

[53] Girard MJA (2011). Suh J-KF, Bottlang M, Burgoyne CF, downs JC. Biomechanical changes in the sclera of monkey eyes exposed to chronic IOP elevations. Invest Ophthalmol Vis Sci.

[54] Coleman DJ, Trokel S (1969). Direct-recorded intraocular pressure variations in a human subject. Arch Ophthalmol Chic Ill 1960.

[55] Tsikripis P, Papaconstantinou D, Koutsandrea C, Apostolopoulos M, Georgalas I (2013). The effect of prostaglandin analogs on the biomechanical properties and central thickness of the cornea of patients with open-angle glaucoma: a 3-year study on 108 eyes. Drug Devel Ther.

[56] Lindsey JD, Kashiwagi K, Kashiwagi F, Weinreb RN (1997). Prostaglandins alter extracellular matrix adjacent to human ciliary muscle cells in vitro. Invest Ophthalmol Vis Sci.

[57] Leite MT, Alencar LM, Gore C, Weinreb RN, Sample PA, Zangwill LM (2010). Comparison of corneal biomechanical properties between healthy blacks and whites using the Ocular Response Analyzer. Am J Ophthalmol.

